# Update on X-ray Microcalorimeter Arrays Based on Thermal MKIDs (TKIDs)

**DOI:** 10.1007/s10909-024-03134-w

**Published:** 2024-05-27

**Authors:** Miguel Daal, W. Hawkins Clay, Majid Mohammad, Benjamin Mazin

**Affiliations:** 1 School of Physics and Astronomy, Tel Aviv University, 69978 Tel Aviv, Israel; 2 Physics Department, University of California Santa Barbara, Santa Barbara, CA 93106 USA

**Keywords:** X-ray, Microcalorimeter, TKID, Thermal MKID

## Abstract

We report progress on the development of x-ray microcalorimeter thermal kinetic inductance detector (TKID) arrays, where each TKID is an independent pixel. Our goal in developing this detector technology is to arrive at high quantum efficiency, high fill factor, large-format, moderate energy resolution x-ray detector array which can be readily scaled to tens of kilo-pixels, to be used as an x-ray imaging spectrograph for astronomy and metrology applications. We discuss the evolution of the design, how it has been driven by fabrication related constraints, and the resulting impacts on detector performance.

## Introduction

The aim of our research is to develop an x-ray detector technology that has moderate energy resolution, $\sim$ 5 eV, over the 0.2–10 keV range with quantum efficiency $\ge$ 90% over the same range, but that can be scaled and multiplexed to achieve arrays possessing tens of kilo-pixels with $\ge$ 75% fill factor. It has already been demonstrated that MKIDs can be multiplexed to get to these pixel counts, [1]. The challenge is doing this with thermal kinetic inductance detectors (TKIDs), which are not as easy to fabricate as MKIDs. The primary application for these detectors is x-ray astronomy,^1^ but we also anticipate they will be attractive for use in metrology applications such as synchrotron beam-line studies and x-ray microanalysis.

The resolution of the TKID can be estimated by comparing the magnitude of the detector response to heat from the x-ray, to all the sources of noise in the system. These include reducible two-level system noise [2] (TLS) quasi-particle generation-recombination noise [3] and readout amplifier noise, and the irreducible statistical fluctuations determining whether the heat makes quasi-particles or is lost to phonons. Our calculations indicate that a resolution of < 5 eV is achievable. Compare this to 0.5 eV at 5.9 keV for mechanically scanned x-ray diffractometers [4, 5] and 2.1 eV at 5.9 keV [6] for transition edge sensors, which are more challenging to scale to > 1 $\times$ 10^4^ pixels. It is to exploit the ease with which they can be scaled up to kilo- and perhaps mega-pixel arrays, that we choose to develop TKID technology.

**Fig. 1:**
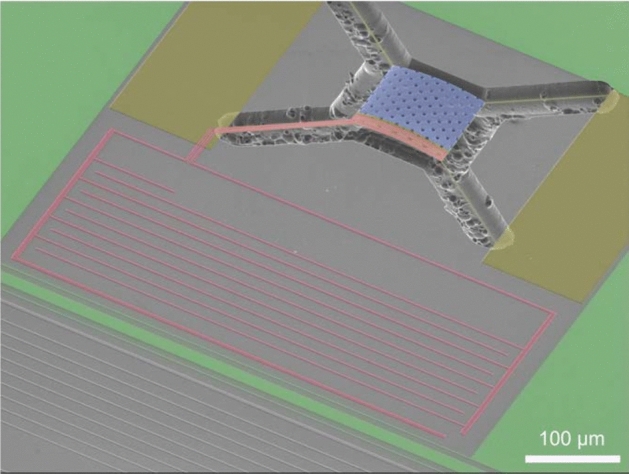
SEM micrograph of XR-7 design microcalorimeter. Color has been added to emphasize the different materials:  is the TiN;  is the SiN;  is the Ta absorber and  is bare Si. The inductor trace runs along the left membrane leg and is meandered in a rectangular block to the lower left of the perforated 500 ${\upmu }\hbox {m}$ thick Ta absorber. The membrane was released using a XeF_2_ etch for the *XR-7* design. This is the design featured in Ulbricht et al. [7] which gave 75 eV at 5.9 keV energy resolution. Reanalysis of the same data improved the energy resolution to 41 eV at 5.9 keV [8] Reprinted from Ulbricht et al. [7]

### Principle of Operation

In our TKIDs, an x-ray absorber is thermally coupled to the inductor of an MKID, while sufficiently isolating this system from the thermal bath. The isolation is accomplished by placing the absorber and the inductor on a thin membrane which has low thermal conductance to the bath. To minimize the thermal quasi-particle population, the bath temperature is limited to $<{T_c}/{8}$, where the $T_c$ is that of the MKID. When an x-ray strikes the absorber, the absorber heats up to a temperature determined by the x-ray energy and the heat capacity of that system, $\delta {T} = {{E}}/{C_\text {tot}}$. The MKID, acting as a thermometer, responds in proportion to the temperature rise of the absorber. This response is in the form of a shift in the transmitted phase and amplitude (a.k.a. dissipation) of the microwave voltage wave exciting the resonator, figure 6. The magnitude of the shift in the transmitted phase and amplitude are recorded and give an estimate of the energy of the x-ray.

### The Absorber Material

There are several considerations that go into the choice of absorber material. Non-metallic absorbers typically have too low a thermal conductivity to uniformly thermalize upon x-ray strike. Additionally, pair creation in a semiconductor absorber also slows down thermalization. Since it must be in close thermal contact with the inductor, which has a microwave frequency current flowing through it, an eddy current may be induced in a metallic absorber. If the absorber is not a superconductor, eddy currents will diminish the resonator quality factor and heat the system. Short quasi-particle lifetimes, Table 1, are needed in these superconducting absorbers to obtain the fast thermalization required for excellent energy resolution. A normal metal on the absorber, but far away from the field of the inductor, can be used to decrease quasi-particle lifetimes.

Usually absorber thicknesses greater than 5 μm are needed to stop 10 keV x-rays, with the precise thickness required depending on the absorber material. This is challenging to vapor deposit, but it can be done. Electrodeposition from aqueous solution would be the preferred deposition method, but few superconductors can be plated in this way. Tantalum (Ta), our preferred absorber material, cannot be electroplated from aqueous solution and > 6$\,{\upmu }\hbox {m}$ of it is needed for the TKID absorbers to achieve the desired quantum efficiency. Vapor deposition of Ta requires attention to the film stress, the film density and to the control of impurities which increase its heat capacity, [9] e.g. hydrogen.

### Approach to Detector Development

It is usually the case that we cannot accurately predict the total effective heat capacity and thermal conductance of the system. Reasons for this include: the membrane heat capacity and thermal conductance are significantly affected by the method used for its release; superconducting tantalum heat capacity in the temperature range of our detector operation are not well specified in the literature; phonon transport simulations of our membranes require more computing power than we have at hand and are unable to predict the dominant mode of scattering leading to a large uncertainty in the conductance estimation. As a result, our detector development approach starts with a very rough estimate of the feature dimensions required to give suitable heat capacity, thermal conductance, as well as other physical detector parameter values that elude accurate prediction (e.g. resonator capacitance, resonant frequencies, TLS noise,...). We then fabricate and test devices with feature dimensions that vary around the estimate values, subject to fabrication constraints (e.g. maximum Ta thickness we can deposit and etch), and iterate our design in the direction of those dimensions which brought us closest to the detector performance goals we have adopted. Consequently, the controlling detector parameters are feature dimensions, materials used and the fabrication techniques. We focus on presenting these parameters throughout the article.

## Previous Designs

Knowledge of our past x-ray detector designs helps to understand the choices we made for later designs. Use of silicon substrates, Ta absorbers suspended on free standing silicon nitride (SiN) membranes, lumped element MKIDs where the capacitor is on silicon and the inductor runs over the membrane, are the only commonalities among all design iterations, past and present.

### The XR-7’s

The *XR-7* design, Fig. 1, was the first of our x-ray TKID designs to give a good enough energy resolution to publish, [7]. The resonators were made entirely of titanium nitride (TiN). The lumped inductor of which was routed down one of the four beams suspending the membrane. On the membrane, the inductor was brought into thermal contact with Ta absorber. The membrane was released using a XeF_2_ isotropic etch. For the XeF_2_ gas to reach under and etch the central portion of the membrane, perforations in the membrane and the Ta absorber were required.

**Fig. 2:**
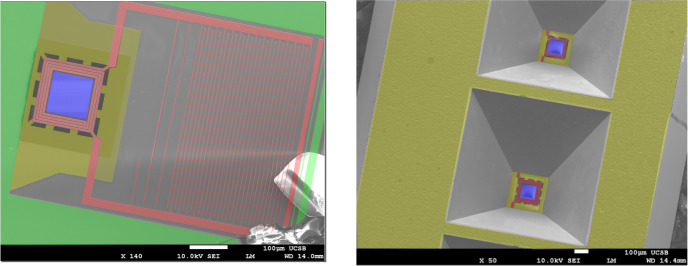
Two SEM micrographs of our XR-8 resonators. Color has been added to emphasize the different materials:  is the WSi;  is the SiN;  is the tantalum absorber and  is bare Si. Left image: top view of the TKID; the inductor trace can be seen running along the upper and lower right membrane legs and wrapping around the Ta absorber. Right image: bottom view of the TKID

The *XR-7’s* exhibited long TKID pulse rise times, $\sim$ 45 $\upmu \hbox {s}$ . This was probably due to the perforations impeding thermal transport and increasing the time needed for the absorber to reach a uniform temperature. Consequently, the TKID response is prone to depending on the location of the x-ray photon strike on the absorber. The energy resolution of the TKID also suffers from the prolonged thermalization time of the absorber because a large fraction of the x-ray energy is lost as heat to the bath over the pulse duration resulting in smaller signal-to-noise. The pulse fall times, ${C_\text {tot}}/{G_\text {tot}}$, were also too long, $\sim$ 500$\,\upmu \hbox {s}$, suggesting that the membrane thermal conductance to the thermal bath, $G_\text {tot}$, was too low. The *XR-7* phase responsivity, ${{\text {d}} \theta }/{{\text {d}} T}$, was too large resulting in easily saturated pulses. The 2D absorber format did not provide a path to achieve our fill factor goal. The TiN resonator material was non-uniform in superconducting state parameters (e.g. T_C_, L_K_ and therefore energy gap $\Delta \approx 1.75 k_B T_c$) across the wafer, and the sputter system used to deposit it became unavailable, forcing us to look for an alternative resonator material.

### The XR-8’s

The *XR-8* design, Fig. 2, replaced the TiN used for the resonators with tungsten silicide (WSi), an amorphous superconductor made in our (replacement) sputter system by co-sputtering tungsten and silicon. In this design iteration, different membrane geometries were explored to find the one with the most favorable conductance and resistance to breakage. The membranes were released by a potassium hydroxide (KOH) silicon etch through the wafer. The KOH etch obviates the need for membrane perforations and allows us to use an absorber containing no holes.

Releasing the membranes in the *XR-8* design proved challenging. Plasma-enhanced chemical vapored deposited (PECVD) SiN was used to encapsulate and protect the feedline, ground plane, resonators and absorber metallization for *XR-7* devices during the XeF_2_ etch. Unfortunately, KOH attacks PECVD SiN making its use unsuitable for the *XR-8* membrane release. Instead, a spin-on resist called Protek made by Brewer Science was used for this purpose. Use of Protek increased resonator yield from about 3% with the PECVD SiN to about 25%. The Protek failed to prevent KOH from attacking metal features close to hole features in the membrane. TKIDs on membranes containing no holes were always fine.

**Fig. 3:**
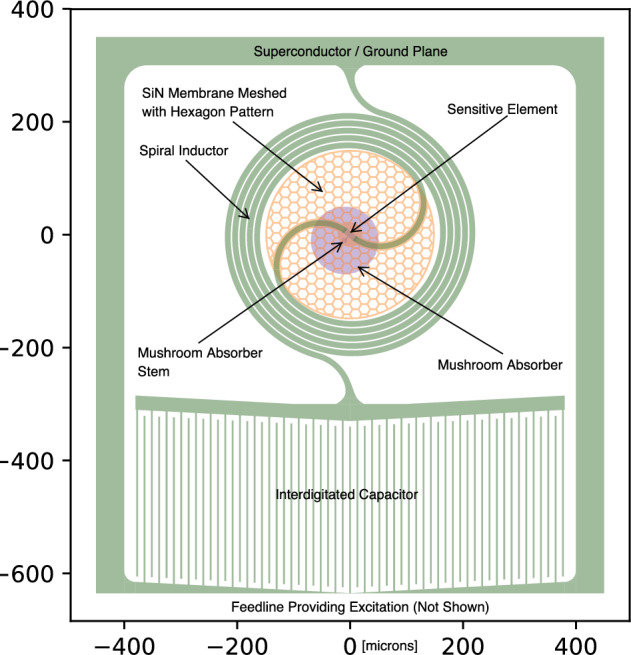
A schematic illustration of the *XR-9* TKID design with various features labeled. Transparency has been used to show overlapping features. Micron distance scales are shown on the axes. A meshed membrane is depicted, but both meshed and solid membranes are on the mask

We quickly found that successfully fabricated (Fig. 2) devices produced unexpectedly small pulses when exposed to a ^55^Fe source. This was the case irrespective of the membrane geometry and the Ta absorber thickness or lack thereof. These symptoms point to the presence of a high heat capacity on the membrane. But for the devices with no absorber, the only material on the membrane is WSi. Additionally, the literature (e.g. Beyer et al. [10]) tells us that KOH released SiN membranes typically have lower heat capacity than XeF_2_ released SiN, which was another driver for our switch to KOH etching. We concluded that the heat capacity of WSi is too high for use on the membrane. We discontinued development on the *XR-8* devices.

**Table 1:** Superconducting transition temperature ($T_{c}$), quasi-particle lifetime ($\tau _{\text {qp}}$) and resistivity ($\rho$) of materials we have used in our TKIDs. Unless indicated, values come from in-house measurements

	$T_{c}$ $[\text{mK}]$	$\tau _{\text {qp}}$ $[\upmu \hbox {s}]$	$\rho \,[\upmu \Omega\, {\text{cm}}]$
WSi	870	11–15	200
TiN	1200	10–200	100
Nb	8500	–	20
Hf	400	50–80	130
$\alpha$-Ta	3700	35	30
$\beta$-Ta	700	35	160

WSi: the W to Si ratio is approximately 1:2.12. *TiN*: these films were made in the sputter system to which we lost access; $\tau _{\text {qp}}$ too long, [11]; mediocre kinetic inductance, $L_{{\text {K}}}$; terrible film uniformity in terms of spatial variation of the superconducting gap parameter. $\beta$-Ta is what naturally grows when sputtering Ta on most surfaces. A $\sim$ 5 nm thick seed layer of Nb is used to grow $\alpha$*-Ta*. $\tau _{\text {qp}}$ for Ta comes from Mazin [12]. See [13] for details on Hf MKIDs

## Current Design

Having demonstrated the TKIDs can work with *XR-7* design, we decided to change course and pursue the larger fill factors accessible by using a cantilevered absorber. A cantilevered absorber is shaped like a mushroom—a slab of x-ray stopping material supported above the substrate by a narrow stem. Ultimately, the entire resonator would fit under the overhang of the slab on the surface of the substrate. However, in the interest in process development, we would not yet implement that compact resonator design. Instead we would increase the size of resonator features to explore the benefits of suppressing TLS noise, one, by driving the resonators at higher readout power and, two, by making the capacitor large [14]. The narrow resonator features of the *XR-7* and *XR-8* designs conspired with the relatively low bifurcation current thresholds of the TiN and WSi to result in resonators that became nonlinear at drive powers low compared to what the readout system can handle. In the interests of improving uniformity of the superconducting films and attaining higher bifurcation thresholds, we would switch to elemental superconductors where we would not need to concern ourselves with small variations in stoichiometry leading to variations in the superconducting state parameters across the wafer, and we would use tapered and/or curved resonator traces to eliminate locations of high current density.

### The XR-9’s

**Fig. 4:**
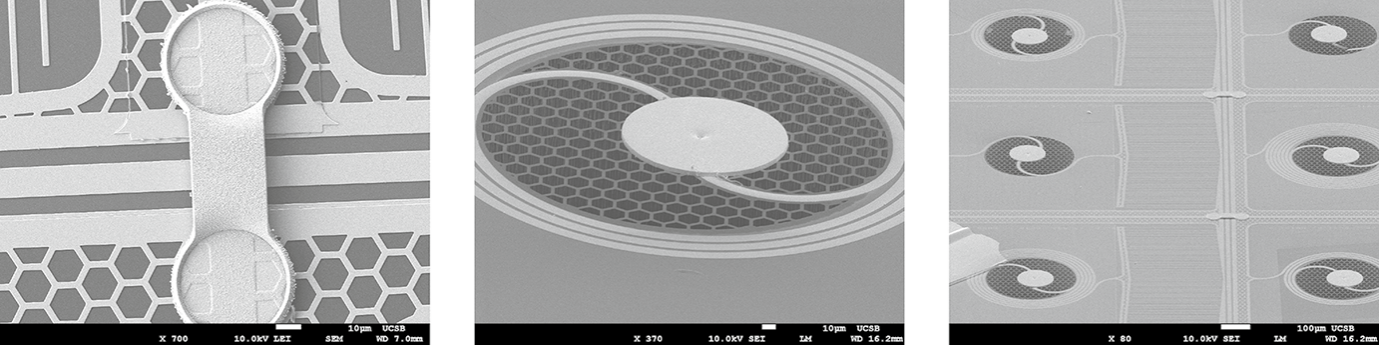
Three SEM micrographs of our XR-9 TKIDs. Left: a micrograph of an airbridge that makes electrical connection between the two grounds of the CPW feedline. It is constructed simultaneously with the cantilevered absorbers, so it is made of the same thick Ta. Center: a micrograph of a $\varnothing$120 $\upmu \hbox {m}$ absorber supported by a $\varnothing$6 $\upmu \hbox {m}$ stem (not visible) constructed on a hexagonally meshed SiN membrane which has been fully released. Right: a micrograph showing six resonators with absorbers on fully released SiN membranes, the feedline and two airbridges crossing it

The *XR-9* design, Fig. 3, implements all the above. Additionally, this design includes airbridges, Fig. 4, to electrically connect the two sides of the CPW feedline ground plane. The resonators are made of two different materials: a high gap, $\Delta$, material comprising most of the inductor, the capacitor, ground plane and feedline, and a low gap, ‘sensor’, material comprising a tiny portion of the inductor which is placed into close thermal contact with the stem of the circular cantilevered absorbers. Each resonator has the same geometry and quantity of metal on its membrane to obtain uniformity of heat capacity and thermal conductance to the bath among TKIDs. The amount of metal on each membrane is minimized to concentrate quasi-particles. Resonant frequencies are tuned by control of the lumped element inductor length off-membrane, as opposed to our usual approach of changing the capacitor area. As a consequence, we expect all the resonators to have the same low value of TLS noise.

Ta has two allotropes we exploit for the *XR-9* TKIDs. Body-centered cubic $\alpha$-Ta is used as the high gap, absorber and airbridge material, and tetragonal $\beta$-Ta is used as the low gap sensor material, Table 1. Originally, we tried using niobium and hafnium, respectively, but hafnium had too low a transition temperature, aversely reacted with the niobium when the two were in contact, and both were incompatible with the piranha cleans we used to remove post-etch residues.

**Fig. 5:**
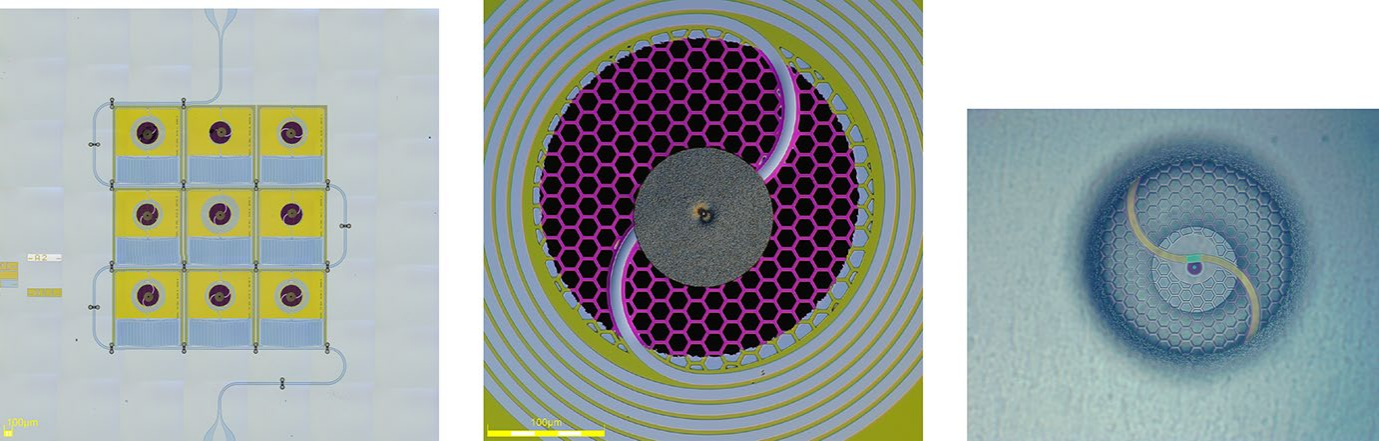
Three optical micrographs of our XR-9 TKIDs. Left: A finished *XR-9* die which was cooled down and used to obtain pulse data. Center: A $\varnothing$120 $\upmu \hbox {m}$ diameter $\alpha$-Ta absorber attached to a meshed silicon nitride membrane by a $\varnothing$6 $\upmu \hbox {m}$ stem. Right: The same Ta absorber as seen from the bottom side of the chip. The $\alpha$-Ta leads appear as gold/amber in color and the $\beta$-Ta sensor appears as teal in color

The *XR-9* fabrication process for the absorbers and the membranes is modeled on that developed by the group at NASA Goddard for their transition edge sensor-based x-ray microcalorimeter arrays, [15]. Our version of this process involves five frontside mask steps and one backside mask step.

**Fig. 6:**
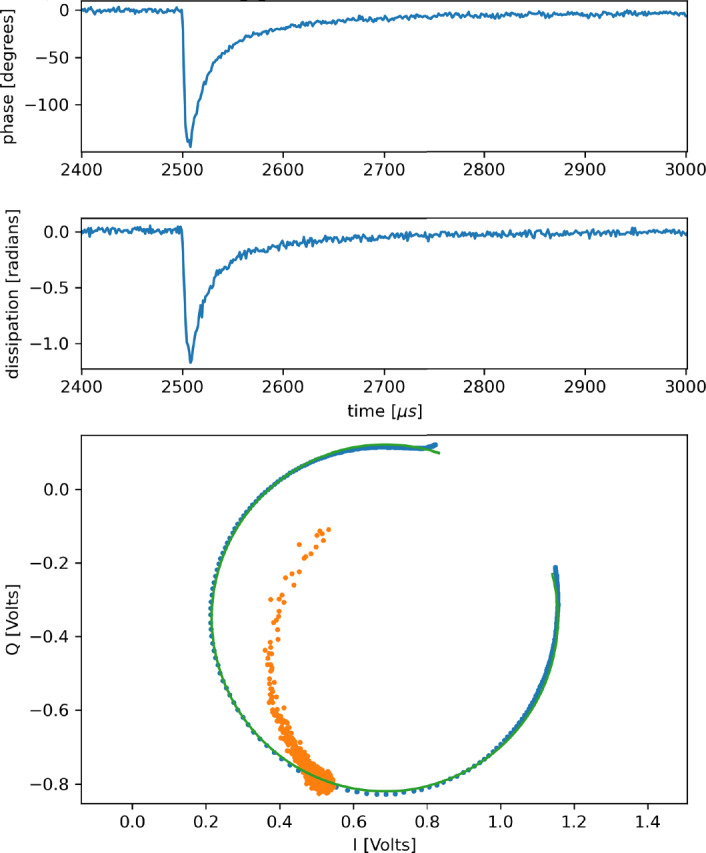
A pulse, most likely due to ^55^Fe from a finished *XR-9* device read out by a traveling wave parametric amplifier, [16]. The resonance loop is at the bottom. The blue dots are the data; the green curve is the fit and the orange dots are the trajectory of the bias with respect to the IQ loop due to the photon strike. Above the resonance loop are traces of the phase angle along the loop and dissipation signal (decrease in loop radius) the pulse traverses along its trajectory. This resonance is at frequency 6.798 GHz, has *Q*_i_ 26,300, *Q*_c_ 14,800 (coupling *Q*) and the device is cooled to < 30 mK

To construct the cantilevered absorbers, a layer of sacrificial photoresist (PR) is spun onto the wafer. The PR is then patterned resulting in the removal of it only in  the locations of the absorber stems and airbridge attachment points. The absorber metal is then sputter deposited on top of the sacrificial PR layer, which has previously been reflowed to obtain sloped edges. The sputter parameters are tuned to give low stress $\alpha$-Ta. The thickness of the sacrificial PR now defines the height of the absorber stems. Another layer of PR is spun on top of the absorber metal and then patterned with the airbridge and (circular) absorber profiles. An ICP RIE etch through the absorber metal and stopping on the sacrificial PR is used to define the airbridges and absorbers. The two PR layers are then dissolved away.

The *XR-9* masks feature solid and meshed membranes to control thermal conductance to the bath. If a membrane is to be meshed, then the mesh pattern is first etched into the SiN on the process wafer front side. Later, regardless of whether the membrane is solid or meshed, the process wafer is bonded to a handle wafer using wax so that its back-side is exposed. Next, a deep RIE Bosch etch through the silicon process wafer releases the membranes and singulates the device die. The vertical directionality of the Bosch etch prevents attack of front-side metal features, solving the problem encountered using KOH. A final acetone soak releases the die from the handle wafer.

### Performance

*XR-9* devices, Fig. 5, were successfully fabricated. They exhibit internal quality factors, Q_i_, upwards of 200,000. Resonator yield across the wafer was $\sim$ 50% on average. Their energy resolution is in need of much improvement.

We are finding that the pulse decay time constants, $\tau = C_\text {tot}/G_\text {tot}$, are ten to twenty times shorter than expected suggesting that the absorber heat capacity, $C_\text {tot}$, is too small or the membrane conductance to the thermal bath, $G_\text {tot}$, is too large, Fig. 6. When we expose the *XR-9* devices to ^55^Fe at a temperature of < 30 mK, we obtain high signal to noise but a very low energy resolution of about 220 eV at 5.9 keV. There are several possible reasons for this. As discussed for the *XR-7’s*, long pulse durations can diminish energy resolution. Another possibility is that the TKID responsively ${{\text {d}}\theta }/{{\text {d}}T}$ is too small leading to a small separation in pulse height between the ^55^Fe lines, which is made indistinguishable by other sources of noise. A revision to the *XR-9* design has been made in attempt to address these problems.

## Conclusion

Over successive design iterations, we have fabricated TKID devices which either give energy resolution of 41 eV at 5.9 keV, or have a fabrication yield and cantilevered absorber design in line with ultimately producing large arrays possessing high fill factor. The remaining tasks are to combine these two successes into one design, and to increase its energy resolution to $\sim$ 5 eV over the x-ray energy range 0.2-10 keV. This will involve improvements to the thermal design of the TKIDs, adjustments to resonator responsivity ${{\text {d}} \theta }/{{\text {d}} T}$, and additional process development to increase yield.

## Data Availability

No datasets were generated or analysed during the current study.

## Footnotes

1 They are a direct response to priorities described in NASA’s 2016 physics of the cosmos technology report (https://pcos.gsfc.nasa.gov/technology/documents/PCOS2016PATR.pdf). This report called out fast, low-noise, megapixel x-ray imaging arrays with moderate resolution as a Priority 1 goal, and large-format, high-spectral- resolution, small-pixel x-ray focal plane arrays as a Priority 2 goal.
